# Culture-induced changes in mRNA expression levels of efflux and SLC-transporters in brain endothelial cells

**DOI:** 10.1186/s12987-020-00193-5

**Published:** 2020-04-22

**Authors:** C. Goldeman, B. Ozgür, B. Brodin

**Affiliations:** Department of Pharmacy, Faculty of Health and Medical Sciences, University of Copenhagen, Universitetsparken 2, 2100 Copenhagen, Denmark

**Keywords:** Blood–brain barrier, Brain endothelium, qPCR, In vitro culture, Co-culture, SLC transporters

## Abstract

**Background:**

The complexity of the neurovascular unit (NVU) poses a challenge in the investigations of drug transport across the blood–brain barrier (BBB) and the function of the brain capillary endothelium. Several in vitro models of the brain capillary endothelium have been developed. In vitro culture of primary endothelial cells has, however, been reported to alter the expression levels of various brain endothelial proteins. Only a limited number of studies have addressed this in detail. The aim of the present study was to investigate mRNA levels of selected BBB transporters and markers in in vitro models of the BBB based on bovine primary endothelial cells and compare these to the levels estimated in freshly isolated bovine brain capillaries.

**Methods:**

Brain capillaries were isolated from bovine cerebral cortex grey matter. Capillaries were seeded in culture flasks and endothelial cells were obtained using a brief trypsinization. They were seeded onto permeable supports and cultured in mono-, non-contact- or contact co-culture with/without primary rat astrocytes. mRNA-expression levels of the selected BBB markers and transporters were evaluated using qPCR and monolayer integrity of resulting monolayers was evaluated by measuring the transendothelial electrical resistance (TEER).

**Results:**

The capillary mRNA transcript profile indicated low expression of *ABCC1* and *CLDN1*. The mRNA expression levels of *TPA*, *OCLN*, *ABCB1*, *SLC2A1*, *SLC16A1* and *SLC7A5* were significantly decreased in all culture configurations compared to freshly isolated bovine brain capillaries. *ALP*, *VWF*, *ABCC1* and *ABCC4* were upregulated during culture, while the mRNA expression levels of *F11R*, *TJP1*, *CLDN5*, *CLDN1* and *ABCG2* were found to be unaltered. The mRNA expression levels of *VWF*, *ALP*, *ABCB1* and *ABCC1* were affected by the presence of rat astrocytes.

**Conclusion:**

The endothelial mRNA transcript profile in bovine capillaries obtained in this study correlated nicely with profiles reported in mice and humans. Cultured endothelial cells drastically downregulated the mRNA expression of the investigated SLC transporters but maintained expression of efflux transporter and junctional protein mRNA, implying that the bovine in vitro BBB models may serve well to investigate basic barrier biology and in vivo permeation of passively permeating compounds and efflux transporter substrates but may be less well suited for investigations of SLC-mediated transport.

## Introduction

The endothelium of the small brain capillaries makes up the blood–brain barrier (BBB). Together with pericytes and astrocytic end-feets, they serve as a restrictive barrier for passage of a wide range of structurally diverse compounds, including most drug compounds. This function is due to the presence of tight junctions (TJs) between the adjacent endothelial cells, efflux transporters and metabolizing enzymes (for references, see [1]). The trans-endothelial transport of nutrients and micronutrients occurs via uptake transporters (solute carriers) and receptors, that are highly regulated.

Several in vitro models of the BBB with brain capillary endothelial cells grown on permeable supports have been developed. These models are widely used to investigate drug transport mechanisms across the BBB, predict CNS drug exposure and to investigate interactions between the cell types of the neurovascular unit (NVU) [6, 18, 21]. Attempts of developing in vitro models of the BBB have included the use of immortalized cell lines from animal or human origin, primary endothelial cells isolated from animal sources (e.g. murine, bovine or porcine) and recently, human induced pluripotent stem cells that have been differentiated towards a brain endothelial phenotype [17, 22, 23, 35]. In general, immortalized cell lines tend to express a wide range of endothelial marker proteins, but they do, however, exhibit low expression of TJ proteins, resulting in cell culture models with low barrier integrity [15]. In vitro BBB models based on isolated primary cells retain many important properties of the BBB by expressing BBB-specific marker proteins, TJ proteins, receptor systems and efflux transporters upon cultivation on permeable supports. There are, however, reports of suboptimal expression of transport proteins, especially of the solute carrier (SLC) family, as recently reviewed by Helms et al. [15]. In the present study, we investigated mRNA levels of selected marker proteins from cultured bovine capillary endothelial cells, and compared those to the mRNA levels in freshly isolated brain capillaries in order to probe the effect of in vitro culture. We furthermore investigated whether important BBB marker genes, efflux pumps/ABC transporters and transport proteins of the SLC family were differentially expressed in brain capillary endothelial cells cultured as monocultures (MC), non-contact co-culture (NCC) and contact co-culture (CCC) with isolated primary rat astrocytes.

We observed that expression levels of efflux transporters were affected by in vitro culture. *ABCB1* was downregulated in all culture configurations, whereas *ABCC1* and *ABCC4* showed elevated expression levels compared to bovine capillaries. The expression level of *ABCG2* was not altered. Furthermore, we observed a significant decrease in expression levels of SLC transporters, as *SLC2A1*, *SLC7A5* and *SLC16A1* were significantly downregulated in all culture configurations compared to bovine capillaries. TJ protein expression levels were not markedly affected, except for *OCLN* which was significantly downregulated in MC and NCC, compared to capillaries.

## Materials and methods

All materials were obtained from Sigma-Aldrich unless otherwise stated.

### Isolation of primary bovine capillaries and rat astrocytes

Bovine brain tissue was obtained from a local abattoir (Mogens Nielsen kreaturslagteri A/S, Herlufmagle, Denmark). The isolation of bovine brain capillaries was performed using a previously described method [16]. Briefly, the leptomeninges were removed and the grey matter was collected in Dulbecco’s Modified Eagle’s Medium (DMEM) and homogenized. The homogenate was filtered through a 160 µm nylon net filter to collect capillary fragments. The collected capillary fragments were centrifuged for 5 min at 500×*g* and the pellet was resuspended in DMEM supplemented with 10% fetal bovine serum (FBS), 10 ml/L MEM non-essential amino acids (NEAA) (×100), 100 U/mL–100 µg/mL pencillin-streptomycin, 125 µg/mL heparin (DMEM-Comp) and the centrifugation was repeated. Pellets were resuspended in digestive enzyme mix containing 2000 U/mL Collagenase III, 3400 U/mL DNAse I and 900 U/mL Trypsin TRL in DMEM-Comp for 1 h at 37 °C in a water bath followed by filtration through a 200 µm nylon net filter. After centrifugation for 5 min at 500×*g*, the pellets were resuspended in cryo-preservation media consisting of 1:9 DMSO: FBS and cryopreserved for later use. To isolate whole capillaries for the analysis of capillary mRNA expression levels, capillaries were collected before the addition of digestive enzymes. The capillaries were spun down in 16% dextran (M_r_: 450,000–650,000) in DMEM at 2630×*g* for 15 min, followed by an additional 10 min spin at 485×*g*.

Primary rat astrocytes were isolated from 6 days old Sprague–Dawley rat pups, as previously described [20]. Pups were euthanized, and the cerebral cortex removed, followed by a gentle homogenization where the tissue was pressed through 80 µm nylon mesh into astrocyte media DMEM-I + 20% serum, consisting of DMEM-AQ, 1% (v/v) MEM NEAA (×100), 100 U/mL: 100 μg/ml penicillin: streptomycin solution, 2.0 mM l-Glutamine (Sigma G7513) and 20% FBS. The homogenate was then triturated using a steel canula (13 G + 5″). The cell suspension was plated in un-coated T75 culture flasks and cells were cultured for 4 weeks (37 °C, 5% CO_2_). Media was changed two times a week, where Ast-DMEM I + 20% serum was used in the first week, Ast-DMEM I + 15% serum in the second week, and AST-DMEM II + 10% Serum (consisting of Ast-DMEM I, 0.25 mM dBcAMP and 10% FBS) in the remaining 2 weeks. The aspirated medium was collected as during the last 2 weeks of culture. This medium, which was named “astrocyte-conditioned medium” (ACM) was later used during culture of bovine brain capillaries. Astrocytes were dissociated using trypsin–EDTA and resuspended in 9:1 FBS: DMSO and cryopreserved until use. Isolated astrocytes were characterized by immunostaining for Glial fibrillary acidic protein (GFAP) and Filamentous-actin to ensure expression of GFAP and a star-shaped morphology, respectively (data not shown).

### Endothelial cell culture

Primary bovine endothelial cells were used after one passage in three different culture configurations in mono culture (MC) and in co-culture with rat astrocytes either as non-contact or co-contact as previously described [16, 18]. Briefly, frozen primary bovine brain capillaries were seeded onto T75 flasks coated with collagen type IV and fibronectin, and cultured in DMEM-Comp + ACM (1:1) for 4 days followed by a trypsinization of the resulting endothelial cells. The cells were subsequently seeded on permeable supports at a seeding density of 90,000 cells/cm^2^ and cultured in either mono-, non-contact- or contact- co-culture configurations. Co-culture models were prepared by seeding astrocytes on the bottom of the permeable support or directly into the trays at seeding density of 120,000 cells/well, 2 days prior to endothelial cell seeding. Cells were then cultured for 3 days in DMEM-Comp. mixed with ACM at a ratio of 1:1 (MC) or DMEM-Comp (NCC and CCC). After 3 days, the medium was changed to differentiation medium containing DMEM supplemented with 312.5 µM 8-(4-CPT)-cyclic adenosine monophosphate, 0.5 µM dexamethasone, 17.5 µM RO-20-1724 and 50 mM *N*-[tris(hydroxymethyl)methyl]-2-aminoethanesulfonic acid. The cells were cultured for additional 3 days. All trans endothelial electrical resistance (TEER) measurements were performed on the experimental day after 6 days of culture on permeable supports with an Endohm Chamber connected to an Evom^2^ Meter (WPI, Friedberg, Germany) to verify the presence of a barrier and was accepted if a TEER value of 500 Ω*cm^2^ or above was obtained.

### mRNA isolation and cDNA synthesis

mRNA from bovine capillaries was obtained by lysing the capillaries in TriZOL, according to the manufacturer’s instructions. mRNA from endothelial cells grown on permeable supports was isolated by using the GenElute™ Universal Total RNA Purification Kit (A4B0208) or Nucleospin^®^ RNA/Protein kit (Macherey–Nagel, Düren, Germany), according to the manufacturer’s instructions. If the GenElute™ kit was used, the DNAse I kit was used prior to cDNA synthesis. mRNA with concentrations between 0.5 and 1.0 µg/µL were used for the synthesis of cDNA using the High Capacity cDNA Reverse Transcription kit (4368814, Thermo Fischer Scientific), according to the manufacturer’s instructions. The reverse transcription was accomplished by a PTC-200 Thermal Cycler (MJ Research, Quebec, Canada). The synthesized cDNA was stored at − 20 °C until further use.

### Real-time quantitative polymerase chain reaction (qPCR)

The qPCR analysis was performed using a Lightcycler^®^ 96 instrument and the FastStart DNA Master SYBR Green I mixture (Roche, Basel, Switzerland) with Riboloc R1 (Thermo Scientific) as RNAse inhibitor. A mixture of cDNA, primers (in a final concentration of 1 µM of forward and reverse primers), Water (PCR-grade) and master mix was used. Primer design was performed using SDSC Biology Workbench and NCBI and all primers were obtained from Thermo Fischer Scientific. The primer specifications are listed in Table 1. A total of 45 cycles were performed after a pre-incubation of 600 s at 95 °C; each cycle contained a period of 95 °C for 10 s, 55 °C for 10 s and 72 °C for 20 s, followed by a melting curve determination. Primer efficiencies were determined in-house by finding the slope of a ten-fold serial dilution calibration curve. Primer efficiencies were then calculated using the equation E = 10^(−1/Slope)^ [31]. The selected primers had efficiencies between 1.9 and 2.1 and melting curves displaying a single product. Selected genes included tight junction proteins Occludin (*OCLN*), Junction adhesion molecule 1 (*F11R*), Zonula occludens 1 (*TJP1*), Claudin-1 (*CLDN1*) and -5 (*CLDN5*), efflux transporters P-glycoprotein (*ABCB1*), Breast cancer resistance protein (*ABCG2*), Multidrug resistance protein 1 (*ABCC1*) and -4 (*ABCC4*), SLC transporters LAT-1 (*SLC7A5*), GLUT-1 (*SLC2A1*) and MCT-1 (*SLC16A1*), as well as the enzyme Alkaline phosphatase (*ALP*), Tissue plasminogen activator (*TPA*) and von Willebrand factor (*VWF*). A set of three reference genes (*HPRT*-*1*, *GAPDH* and *β*-*actin*) was used to normalize the mRNA expression levels of the genes of interest (see Additional file 1: Figs. S1 and S2 for Ct-values).

**Table 1:** Overview of the investigated genes of interest as well as the used reference genes, their gene symbol, name and primers

Gene symbol	Gene name	PCR efficiency	Product size (Bp)	Primer sequence (5′ to 3′)
HPRT1	Hypoxanthine guanine phosphoribosyl transferase 1	2.03	144	F: CGT GGT GAT TAG CGA TGA TG R: TTC ATC ACA TCT CGA GCC AG
ACTB	β-actin	1.91	426	F: AGG CTG TGC TGT CCC TGT AT R: AGG TAG TTT CGT GAA TGC CG
GAPDH	Glyceraldeyde-3-phosphate dehydrogenase	1.93	102	F: CGA CCA CTT TGT CAA GCT CA R: GGA CCT TAC TCC TTG GAG GC
VWF	von Willebrand Factor	1.92	126	F: TCT CAC GAG ACT GCA ACA CC R: TGA AGT GCC TGT CGT CAA AG
ALP	Alkaline phosphatase	1.98	139	F: ATC GGT ACC TGT TTT GCC AG R: TGA TGA CGT TCT TAG CCA CG
TPA	Tissue plasminogen activator	1.96	103	F: TCT TCT CCG TTC TTT TCC GA R: TGT TGG TGA CGG TCC TGT TA
CLDN1	Claudin-1	2.06	122	F: CCG TTG GCA TGA AGT GTA TG R: CCA TGC TGT GGC AAC TAA AA
CLDN5	Claudin-5	2.06	122	F: CAG AAG TAC GAG CTG GGA GC R: TAC TTC ACC GGG AAG CTG AA
OCLN	Occludin	2.05	143	F: CCG GAA GAT GAA ATT CTC CA R: GTT CCA TAG CCT CTG TCC CA
TJP1	Zonula occludens 1 (Zo-1)	2.08	148	F: CGA CCA GAT CCT CAG GGT AA R: GGA TTC TAC GAT GCG ACG AT
F11R	Junctional adhesion molecule 1 (JAM-1)	1.94	113	F: TGC TGA CCT GCT CAG AGA GA R: GGA AGA GTT GCT GAA GGC AC
ABCB1	P-glycoprotein (Pgp)	1.99	127	F: CGG GAC AGA AAG CTC AGT TC R: TAA TGG CGC AAA ATA CAC CA
ABCG2	Breast cancer resistance protein (BCRP)	2.08	139	F: CCA GGC GTT CAT TCA AAA AT R: GCT CTG TTC TGG ATT CCT GC
ABCC1	Multidrug resistance protein 1 (MRP-1)	2.03	106	F: GTT CCC CTC AAT GCT GTG AT R: TCC CGT TGA GAA TTT CGT TC
ABCC4	Multidrug resistance protein 4 (MRP-4)	1.95	124	F: ATT AAA CGC CTG GAA TGT GC R: GTG CAT TGA ACA GCT CCT GA
SLC2A1	Glucose transporter 1 (GLUT-1)	1.92	139	F: TAC CCC AAG AGG TGG CTA TG R: CTG GTC TCA GGC AAG GAA AG
SLC7A5	L-type amino acid transporter 1 (LAT-1)	1.91	111	F: TAG CCA ATC TGG ATC CCA AG R: TCA AGT AAT TCC ATC CCC CA
SLC16A1	Monocarboxylate transporter 1 (MCT-1)	1.91	143	F: TTT ACG CCT TTG ATT CCC AG R: TCC CTA TTG GTT TCT GTG GG

The primer sequence, PCR efficiency and product size are listed for each of the designed primer sets

*PCR* Polymerase chain reaction, *Bp* base pairs, *F* forward, *R* reverse

### Data treatment and statistics

To convert quantification cycle values (Cq) into normalized relative quantities, we used the 2^−ΔΔCq^ method proposed by Livak & Schmittgen [24], but corrected for the estimated efficiency (E): E^−ΔCq^ [14, 28]. The ratio between the efficiency corrected mRNA expression of the reference genes and the gene of interest were then determined. Statistical tests in this study were performed using GraphPad Prism software (version 7). Data are shown as mean + standard error of the mean (SEM). All experiments were performed in at least three individual passages (n denotes number of passages) originating from different animals, and three technical replicates within each passage (N denotes the number of technical replicates), unless otherwise stated. Groups were compared using an ANOVA test (one way), followed by a Tukey post test. P < 0.05 was considered significant.

For Fig. 5, data on human and murine brain capillary endothelial cells were derived from two transcriptomic databases developed by Betsholtz lab [13, 34] and Barres lab [37, 38], respectively. Data were derived as mean expression levels from the respective database, and the mean expression levels were normalized against the corresponding expression levels of HPRT-1, GAPDH and β-actin reported in databases. In addition, the quantitative expression levels of tight junctions and efflux transporters found in the fresh bovine brain capillaries and in endothelial cells cultured in co-contact configuration (plotted in Figs. 2 and 3) were replotted as mean values alongside with data from the transcriptomic databases for a rough visual comparison. Data from the databases were derived as mean values. The data reported in Betsholtz’ database were generated from two mice (Cldn5-GFP;Cspg4-DsRed genotype) [13, 34]. The murine and human data derived from Barres’ database are from two biological replicates of Tie2–EGFP transgenic mice (n = 2) and from two patients (n = 2), respectively [37, 38].

## Results

**Fig. 1:**
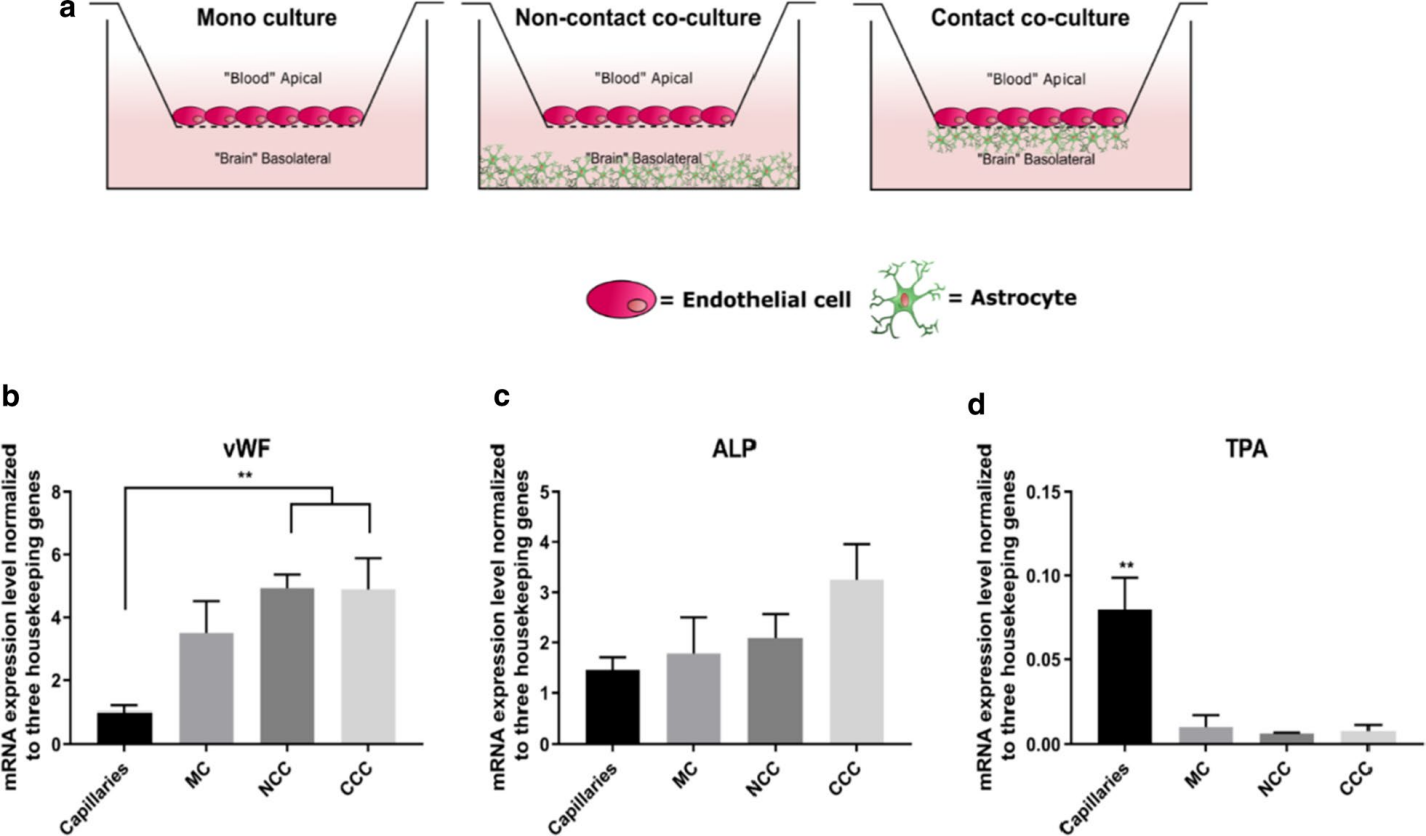
BBB markers. **a** Schematic drawing of the different in vitro culture configurations. Bovine capillary endothelial cells were seeded on the apical side of the filter in all culture configurations. In non-contact co-culture, the rat astrocytes were seeded in the bottom of the well, whereas they were seeded on the basolateral side of the filter in the contact co-culture. **b** Quantitative analysis of the mRNA expression levels of *VWF*. The expression of *VWF* were significantly higher in NCC and CCC compared to capillaries. **c** Quantitative analysis of the mRNA expression levels of *ALP*. There were no significant difference in the expression levels. **d** Quantitative analysis of the mRNA expression levels of *TPA*. All culture configurations were significantly downregulated compared to capillaries. *MC* mono culture, *NCC* non-contact co-culture and *CCC* contact co-culture, *vWF* von Willebrand Factor, *ALP* alkaline phosphatase, *TPA* tissue plasminogen activator. n = 5 − 3, N = 3. **: P ≤ 0.005. Error bars are Mean + SEM

The present study aimed to elucidate to what extent downregulation of mRNA expression of important BBB endothelial genes occurred during culture of in vitro BBB models. Furthermore, it was investigated to what degree co-culture with astrocytes could “rescue” the BBB phenotype in cultured brain capillary endothelial cells, i.e. increase transcript levels which were downregulated in vitro in mono cultures. In vitro mRNA transcript levels were measured in three different culture configurations, monoculture (MC), non-contact co-culture (NCC) and contact co-culture (CCC) (schematic drawing in Fig. 1a) and compared with the mRNA levels in freshly isolated intact brain capillaries.

### The brain endothelial markers von Willebrand factor and alkaline phosphatase were highly expressed in cultured endothelial cells

The mRNA expressions level of three typical brain endothelium markers, von Willebrand factor (*VWF*), Alkaline phosphatase (*ALP*) and Tissue plasminogen activator (*TPA*) were investigated. The in vitro cultures of bovine brain capillary endothelial cells in mono culture and co-culture with astrocytes were compared to that estimated in intact brain capillaries. *VWF* was found to be highly expressed in all in vitro model configurations, and significantly higher mRNA expression levels were observed in endothelial cells co-cultured with astrocytes, as compared to the expression level in intact brain capillaries (Fig. 1b). The mRNA expression level of *VWF* in MC showed a clear tendency to increase as compared to intact capillaries, but statistically non-significant. Contrary to *VWF,* the mRNA expression levels of *ALP* did not differ significantly between the different culture conditions and brain capillaries. However, a tendency of mRNA expression elevation was observed in CCC compared to expression levels estimated in MC, NCC and intact brain capillaries, indicating that the presence of astrocytes influenced induced the mRNA expression levels of *ALP* (Fig. 1c). For *TPA*, on the other hand, we observed a significantly lower expression level in all culture configurations as compared to the expression level estimated in bovine brain capillaries (Fig. 1d).

### mRNA levels of tight junction-associated proteins claudin-5, claudin-1, zo-1 and JAM1 were not changed by culture, whereas occludin transcript levels decreased

**Fig. 2:**
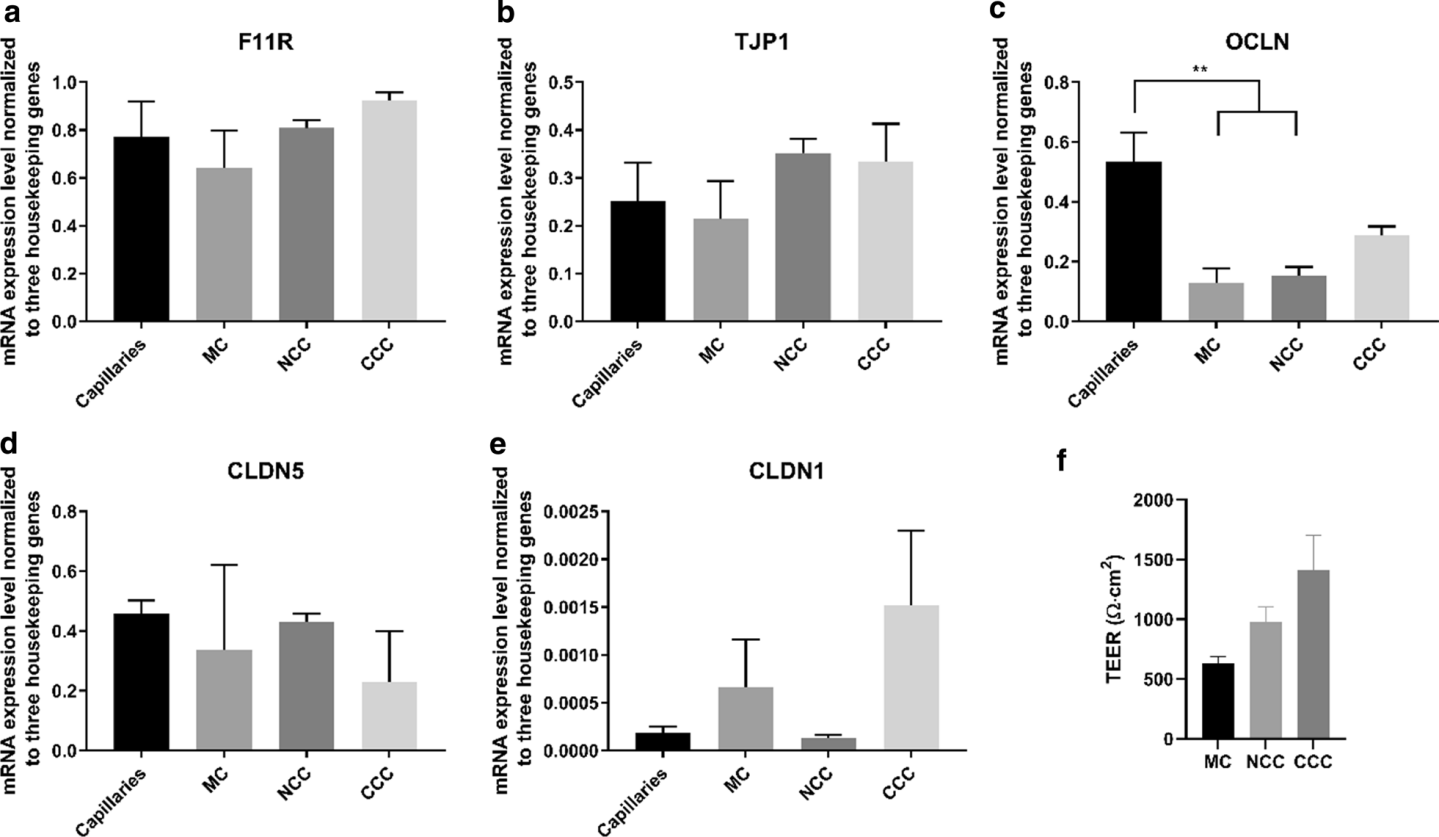
Tight junction proteins. **a** Quantitative mRNA expression levels of *F11R*. There were no significant differences in expression levels. **b** Quantitative mRNA expression levels of *TJP1*. There were no specific differences in expression levels. **c** Quantitative mRNA expression levels of *OCLN*. *OCLN* was significantly downregulated in MC and NCC compared to capillaries. **d** Quantitative mRNA expression levels of *CLDN5*. There were no significant differences in expression levels. **e** Quantitative expression levels of *CLDN1*. *CLDN1* expression levels were low in all culture configurations. n = 3, N = 3. F: Trans-endothelial electrical resistance (TEER) in the different models, n = 4 − 6, N = 4 − 17. Please note that the y-axis are different in all graphs. F11R: JAM-1, TJP1: Zo-1, OCLN: Occludin, CLDN5: Claudin-5, CLDN1: Claudin-1, MC: Mono culture, NCC: Non-contact co-culture, CCC: Contact co-culture. *: P ≤ 0.05, **: P ≤ 0.005. Error bars are mean + SEM

Tight junction proteins (TJs) are highly expressed in the brain capillary endothelial cells resulting in an extremely tight and polarized barrier that selectively controls trafficking of molecules between the blood and CNS. TJs are complex structures that rapidly undergo alterations in expression levels. We measured the transcript levels of the brain endothelial TJ proteins Claudin-1 and -5, Occludin and Zo-1 (*CLDN1*, *CLDN5, OCLN* and *TJP1*) and the junctional adhesion molecule 1, JAM-1, (*F11R*) in brain capillary endothelial cells to see whether TJ complex transcripts were downregulated by in vitro culture, and whether the expression levels could be increased by co-culture with astrocytes. The transcript levels of *CLDN5* were similar in capillaries, MC, NCC and CCC (Fig. 2d). *TJP1* and *F11R* transcript levels showed a similar pattern (Fig. 2a, b). These results indicated that transcript levels of these genes were unaffected by in vitro culture or the presence of astrocytes. The expression level of *OCLN* was found to be significantly downregulated in endothelial cells cultured in MC and NCC, as compared to the level estimated in intact brain capillaries. Expression levels of OCLN in CCC were increased as compared to MC and NCC and was not significantly different from expression levels in capillaries (Fig. 2c). *CLDN1* transcript levels were low in capillaries and in all culture configurations (Fig. 2e) and no significant differences were observed, indicating absence of *CLDN1* from capillary endothelial cells, both in vivo and in vitro. The transendothelial electrical resistance (TEER) of the different culture configurations was measured to ensure barrier integrity (Fig. 2f). The cell monolayers exhibited high barrier tightness, with TEER at 632 (118) Ω∙cm^2^, 977 (278) Ω∙cm^2^ and 1410 (506) Ω∙cm^2^ for MC, NCC and CCC, respectively (Fig. 2f).

### ABC-type efflux pumps *ABCB1, ABCG2, ABCC1* and *ABCC4* responded differentially to culture

**Fig. 3:**
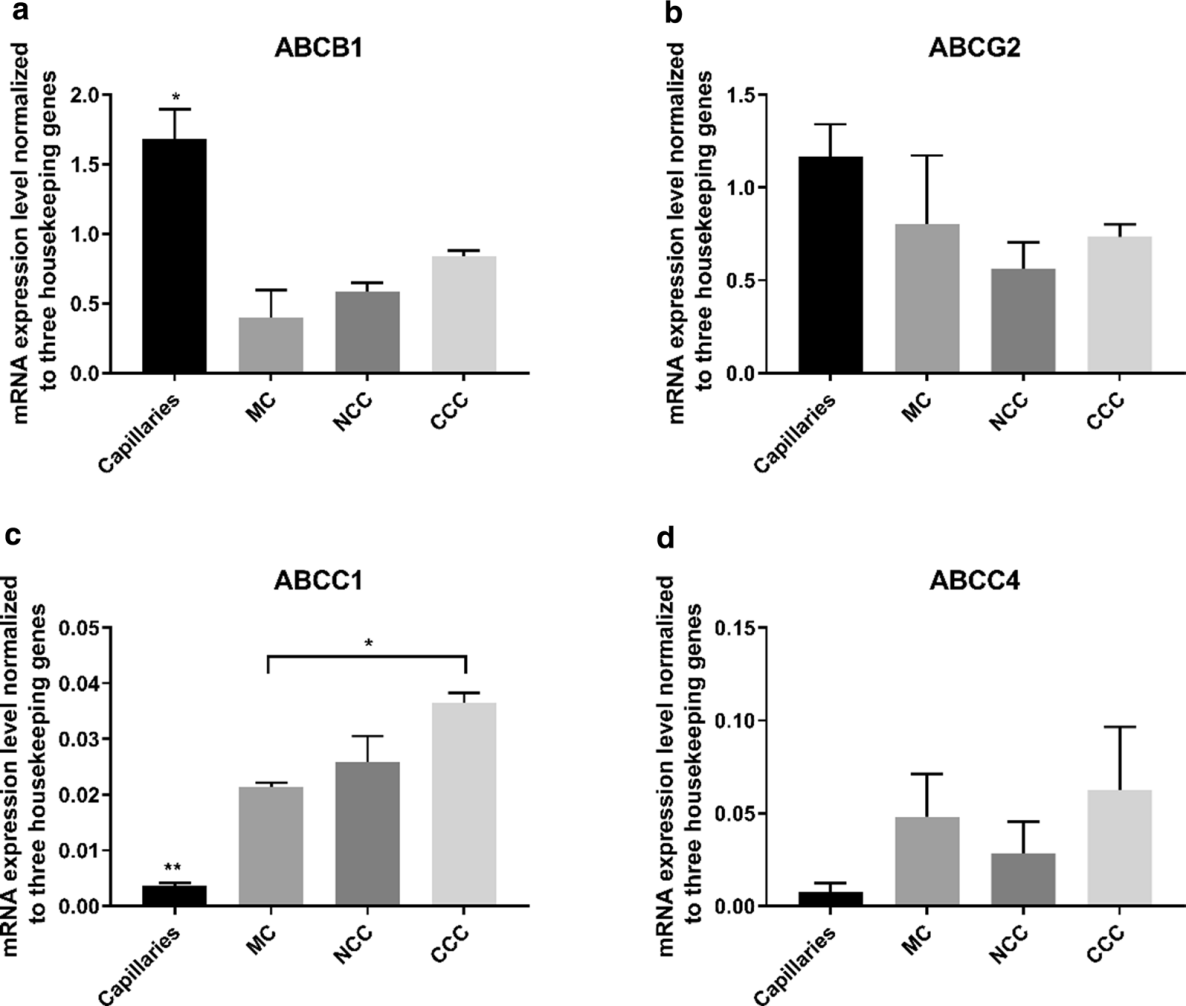
Efflux transporters. **a** Quantitative mRNA expression levels of *ABCB1*. The expression levels were significantly decreased from capillaries to all culture configurations. **b** Quantitative mRNA expression levels of *ABCG2*. There were no significant differences in expression levels of *ABCG2*. **c** Quantitative mRNA expression levels of *ABCC1*. The expression of *ABCC1* was significantly increased from capillaries to all culture configurations. There was a significant increase in the expression in CCC compared to MC. **d** mRNA expression levels of *ABCC4*. There were no significant differences in expression levels, although a non-specific increase was observed from capillaries to all culture configurations. ABCB1: Pgp, ABCG2: BCRP, ABCC1: MRP-1, ABCC4: MRP-4. MC: Mono culture, NCC: Non-contact co-culture, CCC: Contact co-culture. n = 3, N = 3. *: P ≤ 0.5. Error bars are mean + SEM

**Fig. 4:**
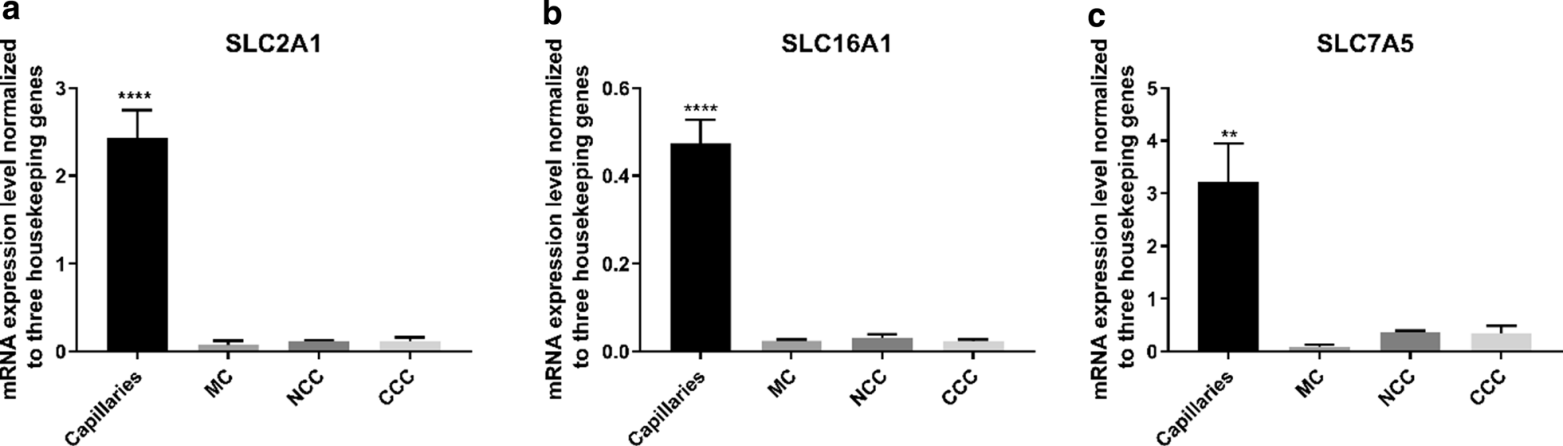
SLC-transporters. **a** mRNA expression level of *SLC2A1*. *SLC2A1* was significantly downregulated in all culture configurations compared to capillaries. **b** mRNA expression level of *SLC16A1*. A significant decrease was observed from capillaries to all culture configurations. **c** mRNA expression levels of *SLC7A5*. *SLC7A5* was significantly downregulated from capillaries to all culture configurations. SLC2A1: GLUT1, SLC16A1: MCT-1, SLC7A5: LAT1. MC: Mono culture, NCC: Non-contact co-culture, CCC: Contact co-culture. n = 3, N = 3. **: P ≤ 0.05, ****: P ≤ 0.0005. Error bars are mean + SEM

Efflux transporters at the BBB represent a major obstacle in drug discovery and development, as many novel small drug candidates developed for brain diseases cannot cross the BBB due to active efflux. We evaluated to what degree the transcript levels of P-glycoprotein (*ABCB1)*, Breast-cancer resistance related protein BCRP (*ABCG2)*, Multidrug-resistance related protein 1, MRP1 (*ABCC1)* and Multidrug-resistance related protein 4, MRP4, (*ABCC4)* were altered in the cell cultures. We found that the expression level of *ABCB1* was significantly downregulated in all in vitro culture configurations as compared to the expression levels found in brain capillaries. Co-culture with astrocytes showed a tendency to increase the expression level of *ABCB1* in bovine brain endothelial cells (Fig. 3a). *ABCG2* exhibited a tendency to decrease in the culture models as compared to the expression level in brain capillaries (Fig. 3b). We observed a significant increase in the expression levels of *ABCC1* in cultures as compared to capillaries. CCCs furthermore displayed higher expression levels than MCs (Fig. 3c). The level of *ABCC4* showed a tendency to increase in culture (Fig. 4d). Comparisons of the absolute expression levels showed that *ABCB1* and *ABCG2* were higher in all culture configurations and in brain capillaries, as compared to *ABCC1* and *ABCC4*.

### SLC transporter transcript levels of LAT-1, GLUT-1 and MCT-1 were markedly lower in cultured endothelial cells, as compared to capillaries

Finally, we evaluated the expression levels of three key SLC transporters at the BBB, namely glucose transporter 1, GLUT-1, (*SLC2A1),* large neutral amino acid transporter 1, LAT-1, (*SLC7A5)* and monocarboxylate transporter 1, MCT1, (*SLC16A1*). A significant decrease in expression levels of *SLC2A1*, *SLC16A1* and *SLC7A5* was observed in the cell cultures compared to mRNA levels in brain capillaries (Fig. 4). The presence of astrocytes did not seem to affect the expression levels of *SLC2A1* or *SLC16A1*. For *SLC7A5,* we observed a non-significantly increase in the expression level in NCC and CCC compared to MC; although it was still significantly lower than the expression level in brain capillaries (Fig. 4c).

## Discussion

In vitro models of the BBB must recapitulate many attributes of the native BBB by forming monolayers with restrictive barrier properties and expressing key proteins, such as TJs, SLC transporters and ABC transporters. Previous studies have shown that in vitro culture of brain capillary endothelial cells may alter the expression levels of important BBB markers and transporters as compared to the expression level in the native brain capillary endothelium [5, 9, 18, 19]. This can have implications for the translational value of data obtained in in vitro experiments when correlating in vitro data to in vivo situations. We therefore aimed at examining the transcript levels of some key genes, known to be highly expressed in the native brain capillary endothelium, in in vitro cultures of brain capillary endothelial cells derived from bovine brains. The endothelial cells were cultured in MC, NCC and CCC configurations with rat astrocytes to examine whether astrocytes could rescue the phenotypic BBB features in the endothelial cells. Rat astrocytes in in vitro cultures of bovine endothelial cells are widely use due to the ability of rat astrocytes to proliferate for an extended culture period (see review: [15], and has been shown to induce several phenotypic BBB traits in bovine capillary endothelial cells [2, 11].

**Fig. 5:**
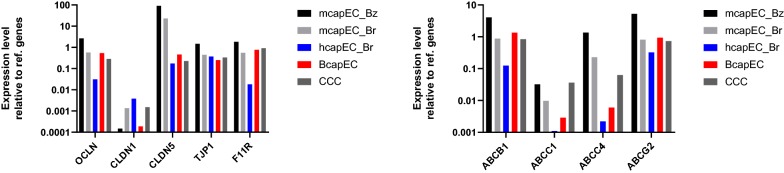
Comparison of TJ and ABC gene expression levels across different species. **a** mRNA expression levels of *OCLN, CLDN1, CLDN5, TJP1* and *F11R,* and **b** mRNA expression levels of *ABCB1, ABCC1, ABCC4* and *ABCG2* in murine, human and bovine brain capillary endothelial cells and in endothelial cells cultured in contact co-culture (CCC) configuration with astrocytes. Transcriptomic data for murine brain capillary endothelial cells (mcapEC) is derived from transcriptomic databases from the Betsholtz lab (Bz) (http://betsholtzlab.org/VascularSingleCells/database.html) and the Barres lab (http://www.brainrnaseq.org/). Data for human brain capillary endothelial cells (hcapEC) are from the Barres lab et al. (Br). All data were normalized against endothelial *HPRT*-*1, GAPDH* and *β*-*actin* expression in the relative databases. Data are shown as mean values. Human data from Barres database: n = 2. Murine data from Barres database: n = 2. Murine data from Betsholtz database: n = 2. Bovine data: n = 3, N = 3

In the present study, we observed that expression of several important BBB genes was altered in the in vitro cultures of primary bovine brain endothelial cells as compared to brain capillaries. *VWF,* a widely used brain endothelial marker, was found to be higher expressed in the in vitro cultures than brain capillaries. A previous study has shown that *VWF* exhibits a protective role in pathological conditions, such as seizures, hypoxia and spontaneous intracerebral hemorrhage by promoting and modulating BBB flexibility and opening during disease states [32]. This protective role of *VWF* indicates that the increased mRNA expression level of *VWF* observed in the in vitro models is presumably due to an in vitro artifact, resulting from adaptation of the endothelial cells to the ambient conditions during in vitro culture. In addition, the expression level of *VWF* was slightly increased in co-culture with rat astrocytes as compared to MC. Several studies have shown that astrocytes in both CCC and NCC have proven efficient in inducing general BBB phenotypic traits in brain capillary endothelial cells [16]. This was also reflected in TEER measurements, where endothelial cells cultured in vitro showed the ability to form a barrier with high functional tightness, and co-culture with astrocytes led to an improved barrier tightness compared to MC. The transcript levels of genes involved in TJ formation were unaltered in the in vitro cultures as compared to those estimated in brain capillaries, except for *OCLN*. The brain capillary endothelial cells were thus still able to form tight monolayers, even though *OCLN* was found to be downregulated in the in vitro cultures. Saitou et al. [29] have previously shown that OCLN is not required for the formation intact TJs in epithelial cells derived from embryonic stem cells lacking OCLN. Similarly, they later found that mice lacking OCLN could still form intact TJs [30]. However, the mice underwent complex abnormalities, such as calcification around brain vessels, indicating another physiological role of OCLN in addition to TJ formation. In a recent study, Berndt et al. [3] showed a loss of TJ complexity in in vitro models compared to in vivo capillaries from human and mouse. They found that other not commonly investigated *CLDN* genes were abundantly present in vivo (*CLDN11*, -*12*, -*25* and -*27*) but almost depleted in vitro. The authors hypothesized that in vitro cultures tend to overexpress *CLDN5*. We did, however, observe unaltered expression levels of *CLDN5* and *CLDN1* in the in vitro cultures and in bovine brain capillaries, recapitulating the in vivo situations. We observed a low baseline expression of *CLDN1* in cultured endothelial cells and capillaries as compared to the expression level of *CLDN5, OCLN, TJP1* and *F11R*. This was in agreement with previous transcriptomic studies on human and murine brain capillary endothelial cells [13, 34, 37, 38], which similarly demonstrated that *CLDN1* was low expressed, while *OCLN, CLDN5, TJP1* and *F11R* were highly abundant in murine and human brain capillary endothelial cells (Fig. 5a). This suggests no or negligible involvement of *CLDN1* in tightening of the BBB. This correlates with previous studies that report an intracellular localization of CLDN1 rather than localization at the junctional zones [3, 27]. The expression level of *ABCB1* was found to be significantly downregulated, while *ABCC1* and *ABCC4* expression levels were increased in the cell culture models. Earlier mRNA transcript studies on bovine endothelial cells have also demonstrated a lower expression of a number of ABC transporters (*ABCG1, ABCA1, ABC2, ABCA3, ABCA7*) in cultures, as compared to capillaries [12].

Helms et al. [17] have previously found that P-gp, BCRP and at least one isoform of MRPs are expressed and functionally active in the bovine capillary endothelial cells co-cultured with rat astrocytes. Previous studies have shown that functional MRP1 is lacking at the BBB in mice [7, 36]. Another study shows that the protein expression level of ABCC1 is under the limit of detection at the porcine BBB [39]. Comparing the data obtained in this study with previous transcriptomic studies on human and murine brain capillary endothelial cells, both *ABCB1* and *ABCG2* are the most dominant members of ABC-transporter coding genes with expression levels being markedly greater than *ABCC1* and *ABCC4* across the species (Fig. 5b).

Taken together, the increases in mRNA levels of *ABCC1* and *ABCC4* are likely due to an in vitro artifact. A similar observation has been reported by Berezowski et al. [2] who showed that MRP1 transcript was present in culture but not in intact bovine capillaries. Another reason for the increase of *ABCC1*, that we observe, may be the use of dexamethasone in culture medium that previously has been reported to induce the expression of functional ABC-transporters in endothelial cells [2, 12]. These findings highlight the difficulty in finding suitable growth factors for enhancing the BBB phenotype in vitro, as the benefits in some cases can lead to in vitro culture artifacts in others, as observed with *ABCC1*. This was also observed in the expression levels of *ALP* between the different culture configurations. A previous study proposed that direct contact between endothelial cells and astrocytes are necessary to maintain a high expression of *ALP* in in vitro cultures [26]. However, we observed that cultured endothelial cells could maintain a high baseline expression level of *ALP,* independent of the culture configurations. The contrasting results suggest that the effects of astrocytes on *ALP* may be lacking due to the presence of cAMP in differentiation media during culture, which similarly to astrocytes has been shown to induce *ALP* in primary porcine brain endothelial cultures [4]. Thus, cAMP may cause the high baseline expression of *ALP* in the cell culture models. In addition to *ABCB1,* the transcript levels of *SLC2A1, SLC16A1* and *SLC7A5* were markedly downregulated by in vitro culture. The downregulation of SLC transporters is a general issue in primary cultures of brain endothelial cells [15]. Calabria et al. [5] have previously shown that in vitro culture of primary rat endothelial cells resulted in a downregulation of SLC transporters such as *Slc2a1* and *Slco1c1* and we have previously shown a similar downregulation of the mRNA expression levels of the insulin receptor and transferrin receptor from capillaries to bovine in vitro models [18, 19]. This possible de-differentiation may pose a challenge for investigations on drug delivery routes involving transporters or receptors that are downregulated in in vitro models. Overall, we thus observed that for the SLCs there was a significant downregulation in cultures as compared to capillaries, which could not be rescued by astrocyte co-culture. It has, however, been shown that bovine pericytes may cause BBB induction as well [33], and we can therefore not exclude that presence of more cell types could increase SLC expression.

It should be noted that the capillary fraction here still contains astrocyte remnants, as previously shown [19]. This may lead to an overestimation of the level of SLC-transporters, which are also present in astrocytes. The findings in the present study emphasize the relevance of investigating expression levels of essential transporters in the BBB. Even though the expression levels were low in our model, it would be very interesting to investigate whether the transporters are active, yielding a potential novel model for investigations of SLC transporters as a drug delivery system. The differences between the mRNA expression in capillaries and our models may largely be a consequence of the environment in vitro that tries to mimic the BBB in vivo. The crude imitation of the microenvironment in vivo, the lack of contact to other cell types, such as pericytes, microglia and neurons, as well as lack of shear stress from blood flow, contributes largely to the differences in in vitro culture (as reviewed by [25]). In fact, the exposure of cells to laminar shear stress is often missing in many in vitro BBB models. Incorporation of shear stress into the culture protocol of in vitro BBB models has previously been shown to promote BBB tightness and to upregulate the transcript levels of efflux transporters and several SLC transporters as compared to the static models [8, 10]. Thus, introducing laminar shear stress to the culture protocol could be another approach for enhancing the phenotypic BBB traits in the in vitro BBB models.

The brain and the NVU are both complex systems to study and are therefore impossible to replicate in vitro, which makes it important to remember possible biases when using in vitro models. Furthermore, it is a field that largely uses primary cells, which are extremely sensitive to changes and the artificial conditions the in vitro models undergo during culture, as shown in the present study. Still, in vitro models are a vital tool in the investigation of potential drug delivery routes across the BBB. Co-culture with primary rat astrocytes had an inducing effect on the mRNA expression levels of some genes, although the overall impact on the model was minor. It is important to remember though, that to validate our results, protein localization and expression studies would benefit this study and highlight the importance of co-culture further. This study showed gene expression alterations of several important transporters in in vitro models of the BBB using primary bovine endothelial cells. It is therefore necessary to thoroughly characterize in vitro models before studying potential drug transport routes across the BBB, as to be aware of the possibility of lower expression of BBB transporters due to culture.

## Conclusion

We investigated differences in mRNA expression levels of selected marker proteins in brain capillaries and brain endothelial in vitro cultures. The present study confirms that the bovine endothelial cell/rat astrocyte co-culture models undergo a phenotypic alteration as compared to endothelial cells in intact brain capillaries. Mono-, non-contact- and contact co-cultures overall retained mRNA expression of junction-associated proteins. *OCLN*, however, was decreased on mRNA expression levels in vitro. The mRNA expression of the SLC transporters *SLC2A1, SLC16A1* and *SLC7A5* were markedly downregulated, and co-culture with astrocytes did not “rescue” the expression levels. Efflux transporters *ABCB1* and *ABCG2* were downregulated while *ABCC1* and *ABCC4* were upregulated compared to in vivo.

At present, no in vitro model of the BBB mimics the native brain capillary endothelium fully. The bovine co-culture in vitro models retain barrier properties and efflux transporter activity but have a suboptimal expression of important SLC transporters which must be considered when investigating trans-endothelial nutrient transport and SLC mediated drug delivery.

## Supplementary information

**Additional file 1: Figure S1.** Quantitative analysis of the mRNA expression levels of the house keeping genes HPRT1, ACTB and GAPDH. Data are shown as mean of cycle quantification value. MC: mono culture, NCC: non-contact co-culture and CCC: contact co-culture. n = 5 − 6, N = 3. *: P ≤ 0.05, **: P ≤ 0.005. Error bars are Mean + SEM. **Figure S2.** Quantitative analysis of the mRNA expression levels of the house keeping genes HPRT1, ACTB and GAPDH. Each of the dots represents the mean of the technical replicates (N = 3) for 5–6 individual batches (n = 5 − 6), as mean of cycle quantification value. MC: mono culture, NCC: non-contact co-culture and CCC: contact co-culture. n = 5 − 3, N = 3. *: P ≤ 0.05, **: P ≤ 0.005. Error bars are Mean + SEM.

## Data Availability

All data used for the publication are available from the corresponding author on request.
