# Supplementary material for: Culture-induced changes in mRNA expression levels of efflux and SLC-transporters in brain endothelial cells
Source: Fluids Barriers CNS. 2020 Apr 22;17:32. doi: 10.1186/s12987-020-00193-5 (PMC7178567; doi:10.1186/s12987-020-00193-5)
Supplement: Supplementary file 1 — Additional file 1: Figure S1. Quantitative analysis of the mRNA expression levels of the house keeping genes HPRT1, ACTB and GAPDH. Data are shown as mean of cycle quantification value. MC: mono culture, NCC: non-contact co-culture and CCC: contact co-culture. n = 5 − 6, N = 3. *: P ≤ 0.05, **: P ≤ 0.005. Error bars are Mean + SEM. Figure S2. Quantitative analysis of the mRNA expression levels of the house keeping genes HPRT1, ACTB and GAPDH. Each of the dots represents the mean of the technical replicates (N = 3) for 5–6 individual batches (n = 5 − 6), as mean of cycle quantification value. MC: mono culture, NCC: non-contact co-culture and CCC: contact co-culture. n = 5 − 3, N = 3. *: P ≤ 0.05, **: P ≤ 0.005. Error bars are Mean + SEM. [file 12987_2020_193_MOESM1_ESM.docx]

# Additional figures for; Culture-induced changes in mRNA expression levels of efflux and SLC-transporters in brain endothelial cells

C. Goldeman, B. Ozgür and B. Brodin*

*Department of Pharmacy, Faculty of Health and Medical Sciences, University of Copenhagen, Universitetsparken 2, DK-2100 Copenhagen, Denmark*

*Corresponding author. [Birger.brodin@sund.ku.dk](mailto:Birger.brodin@sund.ku.dk) , TEL: +45 35336169

**Keywords:**

Blood-brain barrier, brain endothelium, qPCR, *in vitro* culture, co-culture, SLC transporters.

Use of housekeeping genes for normalization of the mRNA values:

Figure S1. Quantitative analysis of the mRNA expression levels of the house keeping genes HPRT1, ACTB and GAPDH. Data are shown as mean of cycle quantification value. MC: mono culture, NCC: non-contact co-culture and CCC: contact co-culture. n=5-6, N=3. *: P≤0.05, **: P≤0.005. Error bars are Mean + SEM.

Mulighed 2:


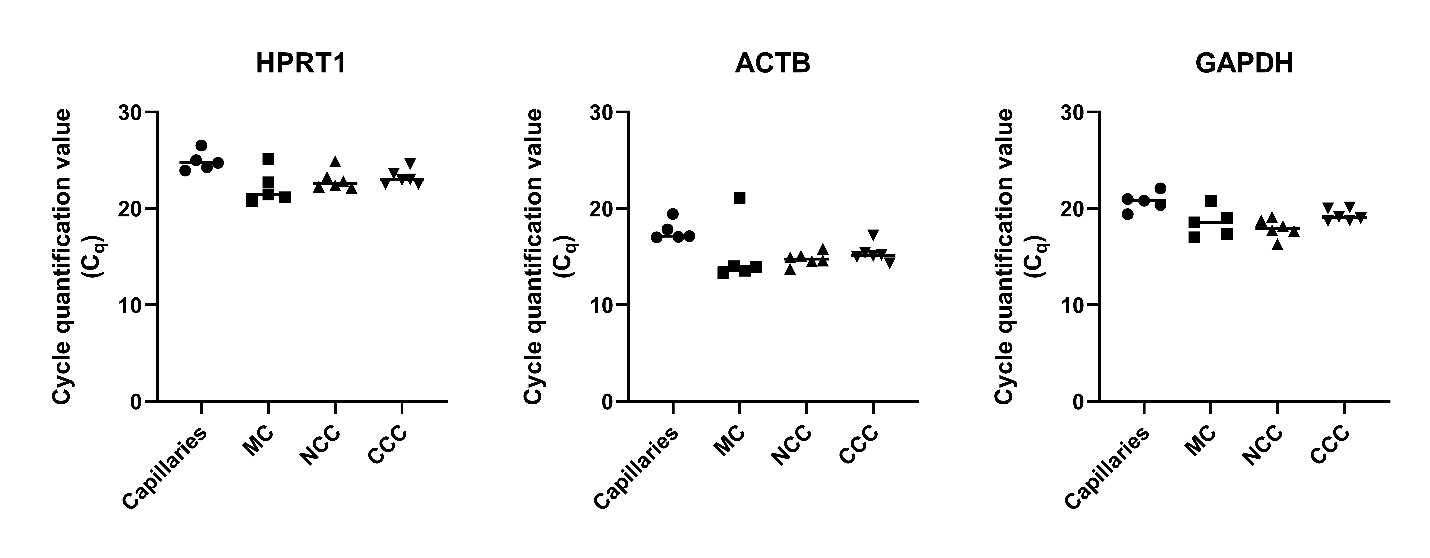


Figure S2. Quantitative analysis of the mRNA expression levels of the house keeping genes HPRT1, ACTB and GAPDH. Each of the dots represents the mean of the technical replicates (N=3) for 5-6 individual batches (n=5-6), as mean of cycle quantification value. MC: mono culture, NCC: non-contact co-culture and CCC: contact co-culture. n=5-3, N=3. *: P≤0.05, **: P≤0.005. Error bars are Mean + SEM.
